# HGIMDA: Heterogeneous graph inference for miRNA-disease association prediction

**DOI:** 10.18632/oncotarget.11251

**Published:** 2016-08-12

**Authors:** Xing Chen, Chenggang Clarence Yan, Xu Zhang, Zhu-Hong You, Yu-An Huang, Gui-Ying Yan

**Affiliations:** ^1^ School of Information and Electrical Engineering, China University of Mining and Technology, Xuzhou, China; ^2^ Institute of Information and Control, Hangzhou Dianzi University, Hangzhou, China; ^3^ School of Mechanical, Electrical & Information Engineering, Shandong University, Weihai, China; ^4^ School of Computer Science and Technology, China University of Mining and Technology, Xuzhou, China; ^5^ Department of Computing, Hong Kong Polytechnic University, Hong Kong, China; ^6^ Academy of Mathematics and Systems Science, Chinese Academy of Sciences, Beijing, China

**Keywords:** microRNA, disease, microRNA-disease association, heterogeneous network, similarity

## Abstract

Recently, microRNAs (miRNAs) have drawn more and more attentions because accumulating experimental studies have indicated miRNA could play critical roles in multiple biological processes as well as the development and progression of human complex diseases. Using the huge number of known heterogeneous biological datasets to predict potential associations between miRNAs and diseases is an important topic in the field of biology, medicine, and bioinformatics. In this study, considering the limitations in the previous computational methods, we developed the computational model of Heterogeneous Graph Inference for MiRNA-Disease Association prediction (HGIMDA) to uncover potential miRNA-disease associations by integrating miRNA functional similarity, disease semantic similarity, Gaussian interaction profile kernel similarity, and experimentally verified miRNA-disease associations into a heterogeneous graph. HGIMDA obtained AUCs of 0.8781 and 0.8077 based on global and local leave-one-out cross validation, respectively. Furthermore, HGIMDA was applied to three important human cancers for performance evaluation. As a result, 90% (Colon Neoplasms), 88% (Esophageal Neoplasms) and 88% (Kidney Neoplasms) of top 50 predicted miRNAs are confirmed by recent experiment reports. Furthermore, HGIMDA could be effectively applied to new diseases and new miRNAs without any known associations, which overcome the important limitations of many previous computational models.

## INTRODUCTION

MiRNAs are one category of short non-coding RNAs (~22nt) which could inhibit the protein production and gene expression through binding to the 3′-UTRs of the target mRNAs at the post-transcriptional and translational level [1–4]. However, miRNAs could also serve as positive regulators according to some studies [5, 6]. In the recent several years, thousands of miRNAs have been detected based on various experimental methods and computational models since the first two miRNAs (Caenorhabditis elegans lin-4 and let-7) were discovered more than twenty years ago [7–10]. There are 26845 entries in the latest version of miRBase, including more than 1000 human miRNAs [11]. Furthermore, accumulating evidences indicated that miRNAs are important components in cells, which could play critical roles in multiple important biological processes, including cell proliferation [12], development [13], differentiation [14], and apoptosis [15], metabolism [16, 17], aging [16, 17], signal transduction [18], and viral infection [14]. Therefore, it is no surprise that miRNAs have close associations with the development, progression, and prognosis of many human diseases [19–24]. For example, the miRNA deregulation is closely related to the development of various cancers [25–28]. Calin et al. firstly clarified that miR-15 and miR-16 are deleted in more than half cases of B-cell chronic lymphocytic leukemia (B-CLL), and this discovery also become the first evidence for the fact that miRNAs are involved in cancer formation [29]. He et al. firstly reported that there are links between the enhanced expression of miR-17 cluster in B-cell lymphomas and the development of c-Myc-induced tumorigenesis [30]. Besides, miR-122 suppresses cell proliferation and tumorigenesis by targeting IGF1R in some breast cancer cases [31]. Experiments further showed that the regulation of Ad6 by miR-122 could significantly improves the safety profile of the whole body after systemic administration, which allows increasing therapeutic doses and therefore improves anticancer efficacy of prostate cancer [32]. Therefore, identifying disease-related miRNAs could effectively promote disease biomarker detection for the treatment, diagnosis and prevention of human complex diseases [33]. Considering vast amount of miRNA-related biological datasets has been generated, it is urgent to develop powerful computational models to predict novel human disease-miRNA associations [34–46].

Many computational methods have been proposed to predict potential miRNA-disease associations based on the assumption that miRNAs with similar functions tend to be related to phenotypically similar diseases [24, 47–51]. Jiang et al. [52] presented a hypergeometric distribution-based computational model to predict novel miRNA-disease associations. This model is mainly based on the integration of disease phenotype similarity network, miRNA functional similarity network, and the known human disease-miRNA association network. Only adopting miRNA neighbor information seriously influences the prediction performance of this model. Shi et al. [53] further proposed a computational model to exploit the functional associations between miRNA and disease by implementing the algorithm of random walk on protein-protein interaction (PPI) network. Considering the assumption that disease tends to be associated with miRNAs whose target genes also have associations with this disease, they paid attentions to the functional links between disease genes and miRNA targets in PPI network by integrating the information of miRNA–target interactions, disease–gene associations, and PPIs. In addition, Mork et al. [54] proposed the computational model of miRPD by integrating protein–disease associations and miRNA–protein interactions to further predict novel miRNA-disease associations. Xu et al. [55] presented an integrated disease-specific miRNA prioritization approach without the rely on known disease-miRNA associations. This method integrates known disease–gene associations and context-dependent miRNA-target interactions. They converted the association probability of a miRNA-disease pair into the functional similarity calculation between the targets of this miRNA and known associated genes of this diseases. However, the predict performances of above several methods were seriously limited by miRNA-target interactions with high false-positive and false-negative results or the incomplete disease-gene association network.

Under the basic assumption that functionally similar miRNAs are regarded to be involved in similar diseases and vice versa, Xuan et al. [56] proposed reliable computational model of HDMP by combining the distribution of miRNAs related with the disease in the k neighbors and miRNA functional similarity to predict the potential disease-related miRNAs. The miRNA functional similarity used in HDMP was integrated by disease phenotype similarity, disease semantic similarity based on the disease terms information content, and known miRNA-disease associations. The important improvement of HDMP over previous studies lies in that it assigned higher weights to members in the same miRNA cluster or family when miRNA functional similarity was calculated. However, HDMP cannot be applied to the new diseases which do not have any known related miRNAs. In addition, HDMP is local network similarity-based computational model, which does not make full use of global network similarity information, which could effectively benefit the prediction performance improvement as demonstrated by many previous studies. Chen et al. [57] proposed the first global network similarity-based computational model, RWRMDA, to predict novel human miRNA–disease associations by considering the information of human miRNA–miRNA functional similarity and known human miRNA–disease associations. The new associations were predicted by adopting the method of random walk on miRNA functional similarity network. RWRMDA has obtained excellent prediction performance based on cross validation and case studies of several important human cancers. However, it also has the important limitation that it could not work for new diseases which do not have any known related miRNAs. Recently, Chen et al. [40] developed a novel computational method of WBSMDA by integrating known miRNA-disease associations, miRNA functional similarity, disease semantic similarity, and Gaussian interaction profile kernel similarity for diseases and miRNAs. WBSMDA could be implemented for the prediction of potential related miRNAs for the diseases which do not have any known related miRNAs and new miRNAs which do not have any known associated diseases. However, the performance of WBSMDA is still not very satisfactory.

Some studies developed machine learning-based computational models to predict novel miRNA-disease associations. For example, Xu et al. [58] constructed a heterogeneous miRNA-target dysregulated network (MTDN) which combines miRNA-target interactions and the expression profiles of miRNAs and mRNAs in tumor and non-tumor tissues. In addition, they performed feature extraction based network topology information and constructed support vector machine (SVM) classifier to identify positive miRNA–disease associations from negative associations. It is well-known that collecting known negative associations is a very difficult and even impossible task. Therefore, inaccurate selection of negative samples would seriously decrease the prediction performance of supervised classifier such as SVM. By integrating disease semantic similarity, miRNA functional similarity, and known miRNA-disease associations, Chenet al. [59] proposed a novel computational model of RLSMDA in the framework of semi-supervised learning to predict potential disease-related miRNAs. RLSMDA could be applied to the diseases without any known related miRNAs. Furthermore, RLSMDA did not need the information of negative miRNA-disease associations. The limitation of RLSMDA lies in the selection of parameter values and the combination of two classifiers in the different spaces.

In this study, we developed a novel computational model of HGIMDA for potential miRNA-disease association prediction. HGIMDA showed superior performance to four classical miRNA-disease association prediction methods (WBSMDA [40], RLSMDA [59], RWRMDA [57], and HDMP [56]). In the case studies of several important human cancers, 45, 44, and 44 out top 50 predicted miRNAs for Colon Neoplasms, Esophageal Neoplasms, and Kidney Neoplasms were verified by recent experimental reports.

## RESULTS

### Performance evaluation

**Figure 1:**
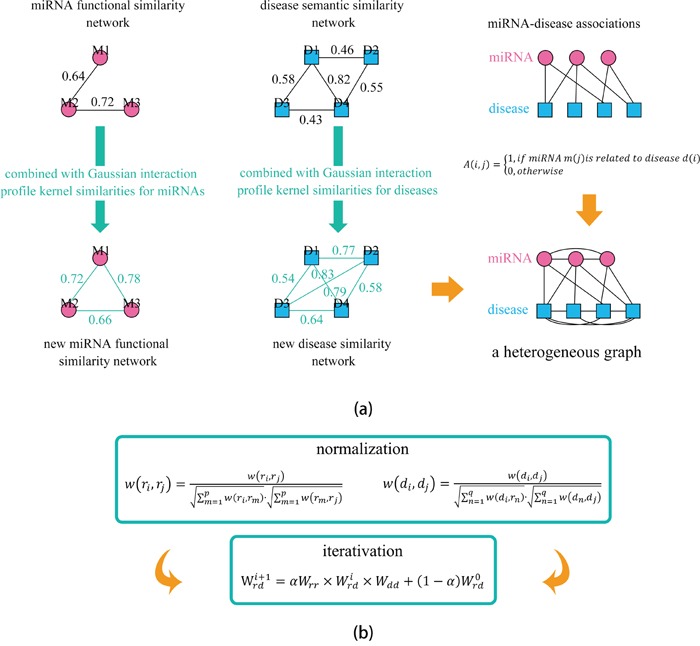
Flowchart of potential disease-miRNA association prediction based on the computational model of HGIMDA **a.** Constructing the heterogeneous graph by integrating miRNA functional similarity, disease semantic similarity, Gaussian interaction profile kernel similarity, and experimentally verified miRNA-disease associations; **b.** Predicting potential miRNA-disease associations based on an iterative equation and obtaining the stable association probability.

We implemented Local and global LOOCV based on the recorded miRNA-disease associations in the HMDD database [60] to evaluate the prediction accuracy of HGIMDA (See Figure 1) and four state-of-the-art computational models for miRNA-disease association prediction: WBSMDA [40], RLSMDA [59], RWRMDA [57], and HDMP [56]. In the validation framework of LOOCV, each known association was treated as test sample in turn and other known associations were used for model training. The difference between local and global LOOCV lies in whether we simultaneously investigated all the diseases. In the local LOOCV, test sample was ranked with the candidate samples composed of all the miRNAs without any known associations with the investigated disease. However, in the global LOOCV, test sample was ranked with all the miRNA-disease pairs without any known confirmed associations. The test samples which obtained ranks higher than the given threshold were considered as successful predictions. Furthermore, we drew Receiver operating characteristics (ROC) curve by plotting the true positive rate (TPR, sensitivity) against the false positive rate (FPR, 1-specificity) at different thresholds. Sensitivity denotes the percentage of the test samples which obtained ranks higher than the given threshold. Meanwhile, specificity denotes the percentage of negative miRNA-disease pairs with ranks lower than the threshold. Area under the ROC curve (AUC) is calculated to demonstrate the prediction ability of HGIMDA. AUC=1 indicates the model has perfect prediction performance; AUC=0.5 indicates the model only has random prediction performance.

**Figure 2:**
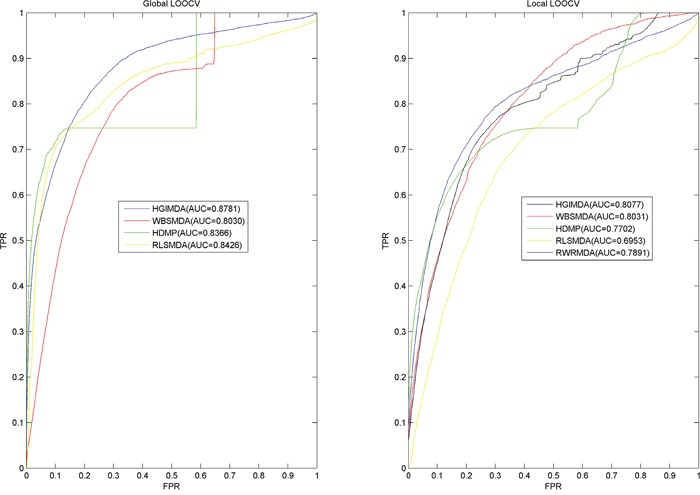
Performance comparisons between HGIMDA and four state-of-the-art disease-miRNA association prediction models (BSMDA, RLSMDA, HDMP, and RWRMDA) in terms of ROC curve and AUC based on local and global LOOCV, respectively As a result, HGIMDA achieved AUCs of 0.8781 and 0.8031 in the global and local LOOCV, significantly outperforming all the previous classical models.

The performance comparisons in the framework of local and global LOOCV have been shown in Figure 2. As a result, HGIMDA, WBSMDA, RLSMDA, HDMP obtained AUCs of 0.8781, 0.8030, 0.8426, and 0.8366 in the global LOOCV, respectively. For local LOOCV, HGIMDA, WBSMDA, RLSMDA, HDMP, RWRMDA obtained AUCs of 0.8077, 0.8031, 0.6953, 0.7702, and 0.7891, respectively. Global LOOCV cannot be implemented for RWRMDA model, for the reason that this model cannot uncover the missing associations for all the diseases simultaneously. In conclusion, HGIMDA has shown reliable and effective prediction performance and potential application value for potential miRNA–disease association prediction.

### Case studies

Here, we further implement case studies of several important human complex diseases to further validate the prediction ability of HGIMDA. Predictive results were verified by checking recent experimental reports from another two databases about miRNA-disease associations, miR2Disease [61] and dbDEMC [62].

Colon Neoplasms is one of the biggest threatens to human life worldwide [63, 64]. Studies show that about half of the Colon Neoplasms patients die of metastatic disease within 5 years from diagnosis [65, 66]. With the rapid development of high-throughput sequencing technologies, researchers have identified several miRNAs associated with Colon Neoplasms. For example, miR-126, which is usually lost in Colon Neoplasms, takes phosphatidylinositol 3-kinase signaling as a target and suppresses neoplastic cells growth [67]. It is also found that miR-145 could inhibit Colon Neoplasms cells growth by targeting the insulin receptor substrate-1 [68]. By implementing HGIMDA to identify potential miRNAs associated with Colon Neoplasms, 10 out of the top 10 and 45 out of the top 50 predicted Colon Neoplasms related miRNAs were confirmed based on miR2Disease and dbDEMC (See Table 1). For example, miR-20a and miR-155 were confirmed to be up-regulated in Colon Neoplasms [69]. MiR-20a and miR-19b shown differential expression between neoplastic conditions and non-tumoral colon tissues [70]. MiR-18a was confirmed to be upregulated in colon cancer tissues which suggested that miR-18a is correlated with Colon Neoplasms [71]. An inverse correlation of miR-21 was found in 10 colorectal cell lines which suggested it is a useful diagnostic biomarker for Colon Neoplasms prognosis [72, 73].

**Table 1:** Here, we implemented HGIMDA to predict potential Colon Neoplasms-related miRNAs

miRNA	Evidence	miRNA	Evidence
hsa-mir-20a	dbDEMC	hsa-mir-106b	dbDEMC
hsa-mir-155	dbDEMC	hsa-mir-143	dbDEMC
hsa-mir-18a	dbDEMC	hsa-mir-200a	unconfirmed
hsa-mir-21	dbDEMC	hsa-mir-9	dbDEMC
hsa-mir-19b	dbDEMC	hsa-mir-1	dbDEMC
hsa-mir-34a	dbDEMC	hsa-mir-15a	dbDEMC
hsa-mir-19a	dbDEMC	hsa-mir-34c	miR2Disease
hsa-let-7a	dbDEMC	hsa-let-7g	dbDEMC
hsa-mir-125b	dbDEMC	hsa-mir-146b	unconfirmed
hsa-mir-221	dbDEMC	hsa-mir-141	dbDEMC
hsa-mir-92a	dbDEMC	hsa-mir-125a	dbDEMC
hsa-let-7b	dbDEMC	hsa-mir-200c	dbDEMC
hsa-mir-146a	dbDEMC	hsa-mir-214	dbDEMC
hsa-mir-29b	dbDEMC	hsa-mir-34b	dbDEMC
hsa-let-7c	dbDEMC	hsa-mir-29c	dbDEMC
hsa-mir-200b	dbDEMC	hsa-mir-101	unconfirmed
hsa-mir-16	dbDEMC	hsa-mir-181b	dbDEMC
hsa-let-7d	dbDEMC	hsa-mir-210	dbDEMC
hsa-mir-199a	unconfirmed	hsa-mir-205	dbDEMC
hsa-mir-29a	dbDEMC	hsa-mir-24	miR2Disease
hsa-let-7e	dbDEMC	hsa-mir-133a	dbDEMC
hsa-mir-223	dbDEMC	hsa-mir-25	dbDEMC
hsa-let-7f	dbDEMC	hsa-mir-132	miR2Disease
hsa-mir-222	dbDEMC	hsa-mir-181a	dbDEMC
hsa-let-7i	dbDEMC	hsa-mir-429	unconfirmed

As a result, 10 out of the top 10 and 45 out of the top 50 predicted Colon Neoplasms related miRNAs were confirmed based on miR2Disease and dbDEMC (1st column: top 1–25; 2nd column: top 26–50).

Esophageal Neoplasms is reported as the sixth-leading cause of deaths related with cancers and the eighth most common cancer worldwide based on the pathological characteristics [74]. The number of male patients is three to four times higher than the number of the female patients [75]. The overall 5-year survival ranges from 15% to 25% [76]. It is suggested that the survival rate could increase to 90% if the tumors could be diagnosed at an early stage [77]. Therefore, the early detection of Esophageal Neoplasms is vital to cancer treatment [78, 79]. There are a lot of miRNAs which have been confirmed to be connected with Esophageal Neoplasms. For example, miR-98 and miR-214 could suppress migration and invasion in human esophageal squamous cell carcinoma by post-transcriptionally regulating enhancer of zeste homolog 2 [80]. HGIMDA was implemented to identify potential related miRNAs for Esophageal Neoplasms based on known associations in the HMDD database. As a result, 9 out of the top 10 and 44 out of the top 50 predicted Esophageal Neoplasms related miRNAs were experimentally confirmed by reports from dbDEMC (See Table 2).

**Table 2:** We implemented HGIMDA to prioritize candidate miRNAs for Esophageal Neoplasms based on known associations in the HMDD database

miRNA	Evidence	miRNA	Evidence
hsa-mir-17	dbDEMC	hsa-mir-30c	dbDEMC
hsa-mir-18a	dbDEMC	hsa-mir-127	dbDEMC
hsa-mir-19b	dbDEMC	hsa-mir-24	dbDEMC
hsa-mir-200b	dbDEMC	hsa-mir-10b	dbDEMC
hsa-mir-125b	dbDEMC	hsa-mir-181a	dbDEMC
hsa-let-7d	dbDEMC	hsa-mir-106a	dbDEMC
hsa-mir-221	dbDEMC	hsa-mir-7	dbDEMC
hsa-let-7e	dbDEMC	hsa-mir-191	dbDEMC
hsa-mir-29b	dbDEMC	hsa-mir-142	dbDEMC
hsa-let-7f	unconfirmed	hsa-mir-20b	unconfirmed
hsa-let-7i	dbDEMC	hsa-mir-18b	dbDEMC
hsa-mir-16	dbDEMC	hsa-mir-195	dbDEMC
hsa-mir-29a	dbDEMC	hsa-mir-30d	dbDEMC
hsa-mir-222	dbDEMC	hsa-mir-182	dbDEMC
hsa-mir-106b	dbDEMC	hsa-mir-199b	dbDEMC
hsa-mir-9	dbDEMC	hsa-mir-30a	dbDEMC
hsa-mir-1	dbDEMC	hsa-mir-194	dbDEMC
hsa-let-7g	dbDEMC	hsa-mir-302b	dbDEMC
hsa-mir-125a	dbDEMC	hsa-mir-15b	unconfirmed
hsa-mir-146b	dbDEMC	hsa-mir-92b	dbDEMC
hsa-mir-218	unconfirmed	hsa-mir-302c	dbDEMC
hsa-mir-429	dbDEMC	hsa-mir-107	dbDEMC
hsa-mir-181b	dbDEMC	hsa-mir-30e	unconfirmed
hsa-mir-132	dbDEMC	hsa-mir-373	dbDEMC
hsa-mir-93	dbDEMC	hsa-mir-219	unconfirmed

As a result, 9 out of the top 10 and 44 out of the top 50 predicted Esophageal Neoplasms related miRNAs were confirmed by experimental reports from dbDEMC (1st column: top 1–25; 2nd column: top 26–50)

Kidney Neoplasm is a nonhomogeneous cancer which accounts for 3% of adult malignancies [81]. There has been an increasing trend for the incidence and mortality rates of Kidney Neoplasm over the past few years. Specifically, more than 250,000 new cases of kidney cancer are diagnosed every year [82]. As the most common form of adult Kidney Neoplasm [83], renal cell carcinoma (RCC) is comprised of several different types of cancer [84–86], including chromophobe RCC (chRCC), collecting duct carcinoma (CDC), clear cell RCC (ccRCC), and papillary RCC (PRCC) [87–89]. Experiments indicated that the histopathology of Kidney Neoplasm has been connected with different genetic changes [90, 91]. Recently, accumulating studies have shown that many miRNAs are associated with Kidney Neoplasms. For example, miR-215, miR-200c, miR-192, miR-194 and miR-141 were downregulated in Kidney Neoplasms [92]. What's more, their common target ACVR2B was found to have strong expression in renal childhood neoplasms [92]. Furthermore, miR-21 was up-regulated in Kidney Neoplasms which corresponds to lower Kidney Neoplasms survival [93]. Finally, we implemented HGIMDA on Kidney Neoplasms for potential disease-related miRNA prediction. As a result, 9 out of the top-10 candidates and 44 out of the top-50 candidates of Kidney Neoplasm related miRNAs were verified by dbDEMC (See Table 3). As for the top 5 confirmed Kidney Neoplasms related miRNAs, miR-17 was found differentially expressed in Kidney Neoplasms compared to normal cell tissues [94]. MiR-20a, miR-155, and miR-18a were found up-regulated in Kidney Neoplasms while miR-145 was found down-regulated.

**Table 3:** We implemented HGIMDA on Kidney Neoplasms for potential disease-related miRNA prediction

miRNA	Evidence	miRNA	Evidence
hsa-mir-17	dbDEMC	hsa-mir-222	dbDEMC
hsa-mir-20a	dbDEMC	hsa-let-7i	dbDEMC
hsa-mir-155	dbDEMC	hsa-mir-200a	dbDEMC
hsa-mir-18a	dbDEMC	hsa-mir-106b	dbDEMC
hsa-mir-145	dbDEMC	hsa-mir-143	dbDEMC
hsa-mir-19b	dbDEMC	hsa-mir-9	dbDEMC
hsa-mir-34a	dbDEMC	hsa-mir-1	dbDEMC
hsa-mir-19a	dbDEMC	hsa-mir-34c	dbDEMC
hsa-let-7a	dbDEMC	hsa-mir-146b	dbDEMC
hsa-mir-125b	unconfirmed	hsa-let-7g	dbDEMC
hsa-mir-126	dbDEMC	hsa-mir-125a	dbDEMC
hsa-mir-221	unconfirmed	hsa-mir-34b	dbDEMC
hsa-mir-92a	unconfirmed	hsa-mir-214	dbDEMC
hsa-mir-146a	dbDEMC	hsa-mir-29c	dbDEMC
hsa-mir-200b	dbDEMC	hsa-mir-101	dbDEMC
hsa-let-7b	unconfirmed	hsa-mir-181b	dbDEMC
hsa-mir-29b	dbDEMC	hsa-mir-205	unconfirmed
hsa-mir-199a	dbDEMC	hsa-mir-210	dbDEMC
hsa-let-7c	dbDEMC	hsa-mir-133a	unconfirmed
hsa-let-7d	dbDEMC	hsa-mir-429	dbDEMC
hsa-mir-16	dbDEMC	hsa-mir-25	dbDEMC
hsa-mir-29a	dbDEMC	hsa-mir-93	dbDEMC
hsa-let-7e	dbDEMC	hsa-mir-181a	dbDEMC
hsa-mir-223	dbDEMC	hsa-mir-24	dbDEMC
hsa-let-7f	dbDEMC	hsa-mir-218	dbDEMC

As a result, 9 out of the top 10 and 44 out of the top 50 predicted Kidney Neoplasms related miRNAs were confirmed by dbDEMC

The results in cross validation and independent case studies exploring on three important human complex diseases have fully indicated the outstanding prediction ability of HGIMDA. Therefore, we further used HGIMDA to prioritize candidate miRNAs for all the diseases investigated in HMDD (See Supplementary Table 1). We anticipate that these prediction results could be confirmed by experimental research in the future.

## DISCUSSION

Recently, more and more researchers start to propose new computational models to search novel miRNA-disease associations. In this paper, considering the hypothesis that functional similar miRNAs are likely to be involved in similar diseases and vice versa, we presented the computational model of HGIMDA to predict new human complex diseases related miRNAs by integrating Gaussian interaction profile kernel similarity, disease semantic similarity, miRNA functional similarity, and known miRNA-disease associations into a heterogeneous graph. The excellent performance of HGIMDA has been demonstrated by the reliable results from both case studies and cross validation of Colon Neoplasms, Esophageal Neoplasms and Kidney Neoplasms. It could be anticipated that HGIMDA can serve as an effective tool for predicting potential miRNA-disease associations, and will be helpful in human disease prevention, treatment, diagnosis, and prognosis.

The reasons of reliable performance of HGIMDA may come from the following several factors. Firstly, the success of HGIMDA is mainly dependent on the integration of several reliable biological datasets into a heterogeneous graph. Especially, the number of known miRNA-disease associations used in this method significantly increases compared with known associations used for previous methods. Secondly, similar to the process of random work, HGIMDA is an iterative process to find the optimal solutions based on global network similarity information, whose improvement over local network-similarity-based models has been fully indicated by the previous studies. However, there are essential differences between HGIMDA and traditional random walk. Traditional random walk set the initial probability vector only based on known related miRNAs with the investigated disease. Therefore, when this disease has no known related miRNAs, random walk can't work. Here, various disease similarity measures, various miRNA similarity measures, and known miRNA-disease association were combined to implement prediction, which ensures that HGIMDA could be used to predict related miRNAs for new diseases which have no known related miRNAs and miRNAs without any known associated diseases. Therefore, the application scope of classical random walk has been significantly broadened. This distinct advantage overcomes the important limitations of many previous computational models. Furthermore, HGIMDA could effectively uncover the missing miRNA-disease associations for all the diseases simultaneously. Limitations also exist in this method. Firstly, the known miRNA-disease associations with experimental evidences are still insufficient. By integrating more available biological information in the future, the prediction performance of HGIMDA could be further improved [95–97]. Secondly, HGIMDA may cause bias to miRNAs which have more associated disease records. Finally, the selection of the parameter value in formula (11) is still not well solved.

## MATERIALS AND METHODS

### Human miRNA-disease associations

Accumulating biological experiments have produced plenty of miRNA–disease associations. The human miRNA-disease association dataset used in this study was downloaded from HMDD database (June, 2013) [60], including 5430 distinct experimentally confirmed human miRNA-diseases associations about 383 diseases and 495 miRNAs. Adjacency matrix *A* is defined to represent known miRNAs-disease associations. If miRNA *m(i)* is related to disease *d(j)*, the entity *A(m(i), d(j))* is 1, otherwise 0. Furthermore, variables *nm* and *nd* are denoted as the number of miRNAs and diseases in the known association dataset, respectively.

### MiRNA functional similarity

Based on the assumption that miRNAs with similar functions tend to be associated with similar diseases and vice versa [24, 47–49, 56], Wang et al. [48] proposed the method of miRNA functional similarity calculation. We obtained miRNA functional similarity from http://www.cuilab.cn/files/images/cuilab/misim.zip and established miRNA functional similarity matrix *FS* to represent the miRNA functional similarity network, in which *FS(i,j)* is the functional similarity score between miRNA *m(i)* and *m(j)*.

### Disease semantic similarity

The relationships among different diseases can be described as a Directed Acyclic Graph (DAG). Disease *D* can be represented as DAG*(D)=(D,T(D),E(D))*, where *T(D)* represents all ancestor nodes of *D* and *D* itself, *E(D)* represents all direct edges from parent nodes to child nodes. Disease MeSH descriptors were downloaded from the National Library of Medicine (http://www.nlm.nih.gov) [98], including Category A for anatomic terms, Category B for organisms, Category C for diseases, Category D for drugs and chemicals and so on. Here, we selected the MeSH descriptor of Category C to construct disease DAGs. The location of each disease term in the DAG could be decided by the tree number of each MeSH descriptor.

The contribution of disease *d* in DAG(*D*) to the semantic value of disease *D* is defined as follows:

(1) $$\{\begin{array}{c} D_{D}(d)=1\;if\;d=D\\ D_{D}(d)=max\{\Delta *D_{D}(d^{\prime })|d^{\prime }\;\in children\;of\;d\}\;if\;d\neq D \end{array}$$

Here, Δ is the semantic contribution factor. The contribution score for disease *d* is inversely proportional to the distance between disease *d* and *D*. The semantic value of disease *D* could be defined as follows:

(2) $$\text{DV}(D)\;=\;{\sum^{\text{​}}}_{d\in \;T(D)}D_{D}(d)$$

It is obviously that two diseases with larger shared part of their DAGs may have greater similarity score. Therefore, the semantic similarity score between disease *d(i)* and *d(j)* is defined as follows:

(3) $$\text{SS }(d(i),\;d(j))\;=\;\frac{\sum_{t\in }{{}_{T}}_{(i)\cap \;T\;(j)\;}(D_{i\;}(t)\;+\;D_{j}\;(t)\;)}{DV\;(i)\;+\;DV\;(j)}$$

### Gaussian interaction profile kernel similarity

Gaussian interaction profile kernel similarity for diseases are constructed based on the assumption that similar diseases tend to be associated with miRNAs with similar functions and vice versa [24, 47–49]. Binary vector *IP(d(u))* is defined to represent the interaction profiles of disease *d(u)* by observing whether there are known associations between disease *d(u)* and each miRNA or not. Therefore, Gaussian interaction profile kernel similarity of diseases *d(u)* and *d(v)* is defined as follows.

(4) $$KD\left(d(u),d(v)\right)=exp\left(-\gamma_{d}||IP\left(d(u)\right)-IP\left(d(v)\right)|{|}^{2}\right)$$

Here, $\gamma_{d}$ is used for kernel bandwidth control, which is obtained by normalizing a new bandwidth parameter ${\gamma^{\prime }}_{d}$ by the average number of associated miRNAs per disease.

(5) $$\gamma_{d}=\gamma^{\prime }{}_{d}/\left(\frac{1}{nd}\overset{nd}{\underset{u=1}{\sum }}||IP\left(d(u)\right)|{|}^{2}\right)$$

Similarly, Gaussian interaction profile kernel similarity between miRNA *m(i)* and *m(j)* is constructed as follows:

(6) $$KM\left(m(i),m(j)\right)=exp\left(-\gamma_{m}||IP\left(m(i)\right)-IP\left(m(j)\right)|{|}^{2}\right)$$

(7) $$\gamma_{m}=\gamma^{\prime }{}_{m}/\left(\frac{1}{nm}\overset{nm}{\underset{i=1}{\sum }}||IP\left(m(i)\right)|{|}^{2}\right)$$

### Integrated similarity for miRNAs and diseases

Considering that miRNA functional similarity scores do not cover all the miRNAs, we integrate miRNA functional similarity scores and Gaussian interaction profile kernel similarity scores for miRNAs to calculate the new integrated similarity scores. That is to say, for the miRNA pair without known functional similarity score, we use Gaussian interaction profile kernel similarity score as integrated similarity; for the miRNA pair with known functional similarity score, we use the average value of Gaussian interaction profile kernel similarity score and functional similarity score as integrated similarity. Therefore, the integrated similarity between miRNA *m(i)* and *m(j)* is defined as follows:

(8) $$\begin{array}{l} SM\left(m(i),m(j)\right)=\\ \left\{ \begin{array}{cc} \frac{KM(m(i),m(j))+FS(m(i),m(j))}{2} & m(i)\;and\;m(j)\;has\;functional\;similarity\\ KM(m(i),m(j)) & otherwise \end{array}\right. \end{array}$$

Similarly, the integrated similarity between diseases *d(u)* and *d(v)* is defined as follows:

(9) $$\begin{array}{l} SD(d(u),d(v))=\\ \left\{ \begin{array}{cc} \frac{KM(d(u),d(v))+SS(d(u),d(v))}{2} & d(u)\;and\;d(v)\;has\;semantic\;similarity\\ KM(d(u),d(v)) & otherwise \end{array}\right. \end{array}$$

### HGIMDA

We developed the computational model of HGIMDA by integrating miRNA functional similarity, disease semantic similarity, Gaussian interaction profile kernel similarity, and experimentally verified miRNA-disease associations to predict potential miRNA-disease associations. Based on the similar nature of miRNA-disease associations, miRNA similarity, disease similarity, and known miRNA-disease associations could be combined together to predict potential associations. For example, for disease *d* and miRNA *m*, we could define their potential association probability as follows if they have no known associations.

(10) $$\text{P}\left(\text{d,m}\right)=\sum_{i=1}^{nm}\sum_{j=1}^{nd}SM\left(m\left(i\right),m\right)*A\left(m\left(i\right),d\left(j\right)\right)*SD\left(d\left(j\right),d\right)$$

This equation means that we can infer potential association between disease *d* and miRNA *m* by summarizing all paths with the length equal to three. We consider the iteration of above procedure and represent the equation as matrix multiplications. Therefore, the iterative equation could be obtained as follows:

(11) P(i+1)=αSM×P(i)×SD+(1−α)A

Here, α is a decay factor similar to the restart probability in the random walk with restart. According to previous literature [99], association probability matrix *P* will converge when *SM* and *SD* are properly normalized utilizing equation (12) and (13), respectively.

(12) $$\begin{array}{l} SM(m(i),m(j))=\\ \frac{SM(m(i),m(j))}{\sqrt{\sum_{l=1}^{nm}SM(m(i),m(1))\cdot }\sqrt{\sum_{l=1}^{nm}SM(m(j),m(1))}} \end{array}$$

(13) $$\begin{array}{l} SD(d(i),d(j))=\\ \frac{Sd(d(i),d(j))}{\sqrt{\sum_{l=1}^{nd}SD(d(i),d(1))\cdot }\sqrt{\sum_{l=1}^{nd}SD(d(j),d(1))}} \end{array}$$

After some steps, the iteration is stable (the change between P(i) and P(i + 1) measured by L1 norm is less than a given cutoff, here we adopt the cutoff as 10^−6^).

## SUPPLEMENTARY TABLE




