# Supplementary material for: HGIMDA: Heterogeneous graph inference for miRNA-disease association prediction
Source: Oncotarget. 2016 Aug 12;7(40):65257–69. doi: 10.18632/oncotarget.11251 (PMC5323153; doi:10.18632/oncotarget.11251)
Supplement: Supplementary file 1 [file oncotarget-07-65257-s001.pdf]

HGIMDA: Heterogeneous graph inference for miRNA-disease association prediction

SUPPLEMENTARY TABLE

Supplementary Table S1: HGIMDA was implemented to prioritize candidate miRNAs for all the diseases investigated in HMDD

See Supplementary File 1
